# Host epigenetic modifications by oncogenic viruses

**DOI:** 10.1038/sj.bjc.6603516

**Published:** 2006-12-19

**Authors:** J M Flanagan

**Affiliations:** 1 CR-UK Viral Oncology Group, Wolfson Institute for Biomedical Research, Gower Street, University College London, London WC1E 6BT, UK

**Keywords:** oncogenic virus, epigenetics, methylation, histone modifications, p300, CBP, KSHV, HHV8

## Abstract

Epigenetic alterations represent an important step in the initiation and progression of most human cancers, but it is difficult to differentiate the early cancer causing alterations from later consequences. Oncogenic viruses can induce transformation via expression of only a small number of viral genes. Therefore, the mechanisms by which oncogenic viruses cause cancer may provide clues as to which epigenetic alterations are critical in early carcinogenesis.

Epigenetics describes the regulation of gene expression and genomic stability by heritable, but potentially reversible, changes in DNA methylation and chromatin structure. Significant progress has been made towards understanding the complementary functions between the various epigenetic regulators, including DNA methyltransferases, methylated DNA-binding proteins and potential DNA demethylases (such as cytosine deaminases). Histone modifying enzymes interact with other regulators of chromatin structure, such as ATP-dependent chromatin remodelling complexes and the opposing effects of the polycomb group (PcG) and trithorax group (TxG) genes (Esteller, 2006). The mediation of transcriptional repression by microRNAs is also gaining popularity as an epigenetic mechanism potentially disrupted in cancer (Esquela-Kerscher and Slack, 2006).

Transcriptionally active genes in a normal cell are marked by unmethylated promoter CpG islands, histone hyperacetylation and particular histone modifications, such as H3 lysine 4 (H3K4) di- and tri-methylation and H3K79 methylation. Transcriptionally repressed genes are marked by promoter CpG island methylation, histone hypoacetylation and H3K9 and H3K27 methylation. The epigenetic landscape of the genome is markedly different in cancers. A general genome-wide hypomethylation is associated with repetititve DNA sequences in all cancer cells, alongside specific promoter hypermethylation of tumour suppressor genes and hypomethylation of oncogenes (Esteller, 2006). In many cancer types, the most commonly hypermethylated genes are involved in DNA repair (hMLH1, MGMT), the cell cycle (p16INK4a, p15INK4b, p14ARF, SFN), apoptosis (DAPK), cell adherence (CDH1, CDH13) and detoxification (GSTP1) (Esteller, 2006). In addition to DNA methylation alterations, a number of critical histone modifications are considered common epigenetic alterations in cancer, including loss of H4K16 monoacetylation and H4K20 trimethylation and gains in H3K4 di- and tri-methylation, H3K79 methylation and H3 and H4 hyperacetylation (Feinberg *et al*, 2006).

As with many genetic changes in cancer, it is difficult to differentiate cause from consequence (Baylin and Ohm, 2006). Moreover, it is unclear whether genetic or epigenetic changes occur first and, if epigenetic, whether these involve DNA methylation or histone modifications. It is very difficult to study early cancer events in tumours or tumour-derived cell lines, because they represent end products of transformation and likely bear little resemblance to the cellular environment of early carcinogenic events (Baylin and Ohm, 2006). The increasingly popular idea that cancers originate from disrupted or ‘cancer-primed’ progenitor cells is supported by the hypothesis that epigenetic disruption of key genes occurs at the earliest stage of cancer development (Feinberg *et al*, 2006). Some of the most convincing evidence for epigenetic disruption of progenitor cells derive from the ubiquitous nature of genome-wide hypomethylation in almost all cancers, common hypermethylation of genes, such as p16INK4a, in many cancer types (and, occasionally, in surrounding normal tissues) and ‘germline epimutations’, such as heightened loss of imprinting (LOI) of insulin-like growth factor 2 in patients at risk for colorectal cancer (Feinberg *et al*, 2006). Such observations suggest the exciting prospect that an epigenetic model could elucidate the early carcinogenic events common to all cancers.

The identification and isolation of the subpopulation of cancer progenitor stem cells, most recently in glioblastoma, represents one potential strategy to elucidate the earliest carcinogenic changes (Singh *et al*, 2004). Identification of genetic and epigenetic differences between cancer progenitor cells and the bulk of other cancer cells may establish a causal link to their phenotypic differences. To date, however, the identification of cancer progenitor stem cells has met with limited success for most cancer types.

Another strategy to investigate the early events in cancer is to manipulate and dissect the process by which oncogenic viruses infect cells and initiate transformation *in vitro* and *in vivo*. Viruses are small and tend to encode only the most important genes required for their survival, for example genes for viral persistence and replication, immune regulation and key genes required to trigger transformation. Oncogenic viruses include retroviruses, such as human T-cell lymphotrophic viruses 1 and 2 (HTLV1/HTLV2), the RNA flavivirus, hepatitis C virus (HCV) and DNA viruses, such as human papilloma virus (HPV), hepatitis B viruses (HBV), polyomaviruses ((JC virus (JCV), BK virus (BKV) and simian virus 40 (SV40)) and the *γ*herpesviruses, Epstein–Barr virus (EBV) and Kaposi sarcoma-associated herpesvirus (KSHV). *In vitro*, oncogenic viruses can induce cellular transformation, producing cells capable of uncontrolled proliferation and often tumorigenic in athymic mice. *In vivo*, viral oncogenesis also requires additional cellular alterations that enable the transformed cell to escape host responses, such as the immune system and apoptosis. Furthermore, viruses exploit epigenetic mechanisms, such as DNA methylation of viral genes, that would otherwise illicit an immune response or the increased expression of DNA methyltransferases that results in increased methylation of cellular genes (Tsai *et al*, 2002; Tao and Robertson, 2003). The frequency of hypermethylation of the cell cycle regulating gene p16INK4a in HBV-, EBV-, KSHV- and HPV-related tumours suggests the importance of its inactivation in virally induced cancers (Platt *et al*, 2002; Li *et al*, 2005). This Minireview describes the interactions between viral proteins and epigenetic regulators that occur in the host cell to mediate the methylation and histone alterations required by the virus. The potential involvement of virally induced epigenetic changes in initiating carcinogenesis is discussed alongside the viral genes potentially responsible. If virally induced epigenetic defects mimic the first alterations in cancer, then these would obviously represent the most effective targets for cancer drug design.

## HOST EPIGENETIC CHANGES OWING TO VIRUSES AND VIRUS-ASSOCIATED CANCERS

The cause or consequence conundrum in cancer epigenetics is equally relevant to the epigenetics of viral infection. It is difficult to differentiate an epigenetic change that is directly due to viral infection, due to the host antiviral response or due to a subsequent downstream effect of the transformation process. Do these viruses happen to infect cancer progenitor cells that are already committed to cancer development and are, thus, just along for the ride? Or are cancer progenitor cells more susceptible to viral infection? Important cancer causing changes may be separated from their consequences via the identification of direct interactions between viral proteins and epigenetic regulators (Table 1).

### KSHV as an example

**Figure 1:**
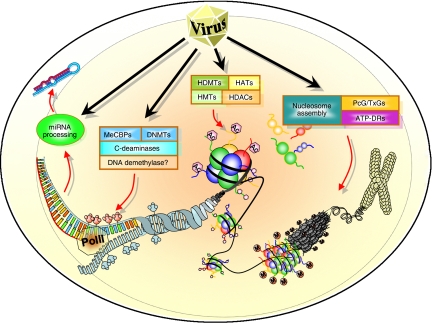
Viral control of the host epigenome. Epigenetic control of gene expression occurs at four different levels starting with chromatin packaging into higher order chromatin structures controlled by ATP-dependent chromatin remodelling complexes (ATP-DRs), PcG and TxG genes. DNA is wrapped around nucleosomes that are assembled from dimers of the histones H2A, H2B, H3 and H4, all of which contain tails that can be either acetylated (HATs) and demethylated (HDMTs) during active transcription or deactylated (HDACs) and highly methylated (HMTs) during repressed transcription. At the nucleotide level, epigenetic control via DNA methylation is mediated by the DNA methylating enzymes (DNMTs), methyl-DNA-binding proteins (e.g. MeCBPs) and cytosine deaminases that could act as demethylating enzymes. Finally, transcriptional repression is also mediated by microRNAs. Oncogenic viruses target DNA methyltransferase activity and p300/CBP histone acetyltransferase activity, but may also target other epigenetic mechanisms to induce carcinogenesis.

Kaposi sarcoma-associated herpesvirus is an oncogenic *γ*-herpesvirus identified as the causative agent of the endothelial tumour Kaposi–sarcoma (KS) and associated with the lymphoproliferative disorders, multicentric Castleman's disease (MCD) and primary effusion lymphoma (PEL) (reviewed in Jenner and Boshoff, 2002). Kaposi sarcoma-associated herpesvirus has both a latent and lytic phase of its life cycle with a more restricted set of genes expressed during latency and in the majority of tumour cells. Particularly in MCD and to a lesser extent in KS and PEL, a small percentage of cells still express lytic genes and maintain lytic replication, which may serve to further spread the virus and maintain important paracrine growth effects of lytic genes such as vGPCR (Jenner and Boshoff, 2002). Kaposi sarcoma-associated herpesvirus contains over 80 open reading frames (ORFs), some of which are pirated from the host genome in order to control proliferation, immune regulation and cell signalling (Jenner and Boshoff, 2002). The virus also employs epigenetic mechanisms, for example chromatin remodelling and demethylation of the lytic switch gene Rta (ORF50) promoter, to control its entry into the lytic phase and hyperacetylation of the latent replication origin to control latent cycle replication (Lu *et al*, 2006). Given this use of cellular epigenetic machinery to regulate its own genome during latency and lytic replication, the virus may also interact with the host epigenome as a deliberate mechanism to alter the cellular environment. There exists clear evidence that KSHV reprogrammes cellular gene expression in both lymphatic and blood vessel endothelial cells, although the mechanism of reprogramming remains unclear (Wang *et al*, 2004). One possibility is that the virus encodes proteins that are involved in DNA methylation, histone modifications, chromatin remodelling or microRNA processing to ensure complete control of cellular gene expression (Figure 1).

Regarding cellular methylation by KSHV, only *p16INK4a* hypermethylation has been identified in the majority of KSHV-infected PEL cell lines and, potentially, in primary PEL samples (Platt *et al*, 2002). This is not surprising, given that *p16INK4a* is one of the most commonly hypermethylated genes in nearly all cancers. This suggests that, in B cells, at least, KSHV is able to invoke DNA methyltransferase activity and may inactivate other cellular genes via methylation. A direct link between LANA and the *de novo* methyltransferase DNMT3a has been proposed in a study, that reveals that LANA recruits DNMT3a to the chromatin and targets repression of approximately 80 cellular genes, some of which are known targets of epigenetic inactivation in various cancers (Shamay *et al*, 2006). In addition, LANA interacts with the DNA methyl binding protein MeCP2, the mSin3 transcriptional repression complex and the histone methyltransferase SUV39H1, thus enabling numerous roles in epigenetic gene regulation (see Shamay *et al* (2006) for individual references). Regarding histone modification of cellular genes, a number of studies have identified a direct link between the KSHV encoded interferon regulatory factors (viral interferon regulatory factor (IRF) 1, 2 and 3) and the histone acetyltransferase complex p300/CBP (Li *et al*, 2000). The binding of cellular IRF3 to CBP is thought to be a requirement for transcriptional activation of the antiviral cytokine interferon-*β* (IFN-*β*). Accordingly, one may assume that KSHV would evolve a mechanism to disrupt IFN-*β* activation, which it has by direct interaction between vIRF1 and either p300 or CBP. In effect, these interactions inhibit histone acetyltransferase activity and lead to histone hypoacetylation, altered chromatin structure and reduced expression of cytokine genes and, presumably, other genes activated by p300/CBP (Lin *et al*, 2001). Increased DNA methylation and decreased histone acetylation are both associated with gene silencing, which could account for the many genes downregulated after KSHV infection.

The importance of epigenetic regulation for gene expression and the reprogramming of cellular gene expression during viral transformation suggests that other viral genes (latent or lytic) may also be involved in epigenetic control of gene expression. Kaposi sarcoma-associated herpesvirus is relatively unique in that it contains numerous genes with cellular homologues and, by investigating the epigenetic functions of the cellular genes, one may also infer interactions or functions related to epigenetic control by the viral genes. Using this strategy, a number of viral genes appear likely epigenetic regulators, including viral interleukin-6 (IL-6), viral G-protein coupled receptor and the 12 KSHV microRNAs (Table 2). Speculation on viral gene function based on the functions of cellular homologues can provide interesting leads and has proved correct for the viral IRFs, but there exist cases wherein the viral gene does not function in the manner of its cellular homologue. For instance, vIL-6 does not bind to the cellular IL-6 receptor. Rather, it binds only to the gp130 receptor. At present, the potential cellular or viral targets of the KSHV microRNAs exist only as *in silico* predictions, among which are the cytokine signalling regulator *SOCS3*, DNA repair gene *RAD21*, the tumour necrosis factor-*α* (*TNF-α*) and the kinase *MAP3K8*, which is also involved in production of TNF-*α* (Cai *et al*, 2005). Whether or not there are cellular homologues of the viral microRNAs has yet to be explored. Accordingly, whether or not the viral genes described here are actually involved in epigenetic regulation must be investigated experimentally.

### Other oncogenic viruses

Increased DNA methylation activity and decreased histone acetylation activity are not unique properties of KSHV, as other oncogenic viruses also share these properties (Table 1). The related *γ*-herpesvirus, EBV, activates DNA methyltransferase activity by increasing the expression of the maintenance methyltransferase DNMT1 and both *de novo* methyltransferases, DNMT3a and DNMT3b (Tsai *et al*, 2002). The LMP 1 protein is one of the key oncogenic viral proteins of EBV and it is not surprising that this is the protein that alters the DNA methyltransferase activity. The hepatitis B virus HBx protein, BK polyoma virus T-antigen and the adenovirus oncogene E1A all increase the activity of DNMT1 alone (Lee *et al*, 2005; McCabe *et al*, 2006). In both EBV and HBV-related epithelial cancers, this results in increased methylation and suppression of E-cadherin, thereby increasing cell migration (Tsai *et al*, 2002; Lee *et al*, 2005). The HPV acts similar to KSHV in that it appears to increase the *de novo* methyltransferase, DNMT3b protein, in nonsmoking lung cancers, curiously only in females (Lin *et al*, 2005). Although no HPV protein has been identified as the culprit for this increase in DNMT3b, a recent study has now shown that HPV E7-protein increases DNA methyltransferase enzymatic activity by directly interacting with DNMT1 (Burgers *et al*, 2006). The link between JCV and increased DNA methylation is more speculative with an association between the JCV T-antigen expression and the methylator phenotype in colorectal cancer (Goel *et al*, 2006). The simian SV40 virus increases DNA methylation by upregulation of DNMT3b via the large T antigen (Soejima *et al*, 2003). The association between SV40 and human cancers is still contentious; however, this does provide an interesting insight into the possibility that activation of methylation may be conserved among other mammalian viruses. Although methylation of p16INK4a occurs in hepatocellular carcinoma associated with the HCV, no direct interactions have yet been identified.

The ability to alter histone modifications and chromatin structure is also common to many oncogenic viruses including EBV, HPV, adenovirus and HTLV1. The EBV nuclear antigens EBNA 2 and 3c alter histone acetylation by interacting with the p300/CBP complex or with histone deacetylase (HDAC), respectively (Wang *et al*, 2000; Knight *et al*, 2003). Human papilloma virus oncoprotein E6 binds and inhibits the histone acetyltransferase activity of the p300/CBP complex similar to KSHV (Patel *et al*, 1999). This transcriptional repressive activity by HPV is further supported by E7 protein interaction with HDAC1 and the Nurd ATP-dependent chromatin remodelling complex that are also involved in repression (Longworth and Laimins, 2004). The adenovirus transforming protein E1A also interacts with the p300/CBP transcriptional complex to peturb or alter the normal functioning during the cell cycle (Turnell *et al*, 2005). This has been described as one of the key events in the induction of E1A-induced cellular transformation. Finally, the retrovirus HTLV1 Tax protein also interacts with the p300/CBP complex to mediate transcriptional repression (Kwok *et al*, 1996).

It is interesting to note that the EBV proteins that interact with epigenetic regulators (EBNA2, EBNA3c and LMP1) are all latent genes and are not typically expressed in Burkitt's lymphoma, gastric cancer and most nasopharyngeal carcinomas. This may lead to the conclusion that the role of virus-induced host epigenetic changes may be limited in these particular cancers. However, it cannot be ruled out that the initial cancer precursor cells may have harboured the virus in a latent phase in which the host epigenome could be altered. Epigenetic fingerprints such as DNA methylation and histone modifications are mitotically heritable and, thus, even if the progeny cancer cell no longer expresses these latent genes, the epigenetic history of the cell may remain. It is also interesting to observe that the proteins that interact with epigenetic mechanisms, LMP1, LANA, E6 and E7, large T antigen, E1A and Tax, are often described as the viral ‘oncoproteins’ as they are often either oncogenic on their own or essential components of viral transformation. This typically leads to the conclusion that the functions of these proteins, in this case the epigenetic functions, are indeed essential for viral-induced transformation.

Returning to the hypothesis that viral-induced epigenetic changes might mimic the early events in cancers, we can observe from these studies described that disruption or alteration of p300/CBP histone acetyltransferase activity is common to many oncogenic viruses, which suggests that it may be one of the critical early events in viral-induced carcinogenesis. Further evidence for an early involvement of p300/CBP in nonviral cancers can be observed in the increased predisposition to childhood malignancies in Rubinstein–Taybi syndrome, which is characterised by germline mutation of CBP as well as numerous somatic mutations in colorectal, breast and gastric carcinomas (Iyer *et al*, 2004). This suggests that abrogation or peturbation of the histone acetyltransferase activity of p300/CBP may also be one of the critical early events in all cancers. Similarly, an increase in DNA methyltransferase activity might also be a crucial early event. Disruption of either the maintenance methylatransferase, DNMT1, or the *de novo* methyltransferases, DNMT3a or 3b, suggests that each oncogenic virus may have evolved different routes to the same goals (methylation of key tumour suppressor genes) or perhaps that different viruses require inactivation of different genes via different methyltransferases. Interestingly, ranid herpesvirus 1 (RaHV-1), which is the aeitiological agent of kidney cancers in the North American leopard frog *Rana pipiens* encodes its own DNA (cytosine-5) methyltransferase, further suggesting that alteration of methylation in the host genome is a highly conserved function in oncogenic viruses (Davison *et al*, 1999). Again, in nonviral cancers, this is also a common finding with an overall increase in DNA methyltransferase activity in many tumours compared with normal tissues (Esteller, 2006). Both of these activities, disruption of histone acetylation and increased DNA methylation, are related to repression of transcription, which provides support for the idea that the critical early carcinogenic events begin with the inactivation of tumour-suppressor genes (Feinberg *et al*, 2006). A more complete understanding of the interactions between viral proteins and epigenetic regulators may be a very important step in understanding the earliest alterations in cancer. In addition, identification of the particular changes induced by viruses might provide novel, and more effective, targets for cancer therapies.

## CONCLUSIONS

It is apparent from current knowledge that KSHV and other oncogenic viruses increase activity of DNA methyltransferases and can decrease p300/CBP-mediated histone acetylation, which are both likely requirements for the inactivation of tumour-suppressor genes. The fact that both of these events also occur in many other nonviral cancers suggests that these may indeed be some of the earliest alterations in carcinogenesis. Identification of cancer progenitor cells with increased DNA methyltransferase activity and abrogated p300/CBP histone acetyltransferase activity would further support this hypothesis. Furthermore, there exists the potential for other viral genes in epigenetic control, as proposed in this review for KSHV, and thus there is scope for identification of novel epigenetic alterations as early carcinogenic events.

There is gathering momentum for the use of epigenetic related therapies, such as HDAC inhibitors, for example suberoylanilide hydroxamic acid (SAHA), which is currently in Phase III trials and has shown reasonable efficacy in haematological and solid tumours. At present, five trials are investigating the use of HDAC inhibitor valproic acid (VPA) in Kaposi sarcoma, EBV-related nasopharyngeal carcinoma and non-Hodgkin's lymphoma, as well as in HIV-infected patients (http://clinicaltrials.gov/). Some circumspection is required, however, because histone deacetylase inhibitors, including valproate, may induce lytic replication, as has been shown for KSHV and EBV (Klass *et al*, 2005; Feng and Kenney, 2006; Lu *et al*, 2006). For an effective drug, it would be preferable to induce lytic replication and induce apoptosis without increasing the viral load. A recent study has shown that valproate can induce entry into the lytic cycle in KSHV-infected PEL cells and can significantly increase production of the virus, leading to cell death by apoptosis (Klass *et al*, 2005). With the addition of ganciclovir and PFA, viral DNA replication was blocked without blocking the lytic cascade and apoptosis, which advocates the use of HDAC inhibitors in combination therapies rather than as single agents in virus-associated cancers. Other HDAC inhibitors (depsipeptide, SAHA, MS-275 and trichostatin A) have also been tested in PEL cells with minimal (3–14%) reactivation (Niedermeier *et al*, 2006). Given our present understanding of the epigenetic control required by oncogenic viruses in the transformation process, the use of HDAC inhibitors, or other epigenetically targeted therapies, is warranted in virus associated tumours.

## Figures and Tables

**Table 1:** Epigenetic interactions of oncogenic viral proteins

**Virus**	**Human Neoplasms**	**Viral Proteins**	**Epigenetic interactions**	**References**
*DNA viruses*				
KSHV	KS	LANA	• Activates DNMT3a	(Li *et al*, 2000; Shamay *et al*, 2006)
	PEL		• Interacts with SUV39H1, MeCP2, mSin3, HP1	
	MCD	vIRFs	• Binds and inhibits p300/CBP HAT activity.	
EBV	BL, NPC, HD	LMP1	• Activates DNMTs 1,3a,3b	(Wang *et al*, 2000; Tsai *et al*, 2002; Knight *et al*, 2003)
	Gastric Cancer	EBNA2	• Binds p300 to activate transcription	
	PTLD	EBNA3c	• Interacts with HDACs	
HPV	Papilloma, carcinomas	?	• DNMT3b protein is increased by HPV in females only	(Patel *et al*, 1999; Longworth and Laimins, 2004; Lin *et al*, 2005; Burgers *et al*, 2006)
		E7	• Binds DNMT1 to increase DNA methyltransferase activity	
			• Binds HDACs and Nurd ATP-dependent remodelling complex	
		E6	• Binds and inhibits p300/CBP HAT activity	
HBV	HCC	HBx	• Activates DNMT1	(Lee *et al*, 2005)
SV40	? Osteosarcoma	Large T-Ag	• Activates DNMT3b	(Soejima *et al*, 2003)
	? Mesothelioma			
BKV	? Brain tumours	Large T-Ag	• Activates DNMT1	(McCabe *et al*, 2006)
JCV	? Gliomas	T-Ag	• May induce methylator phenotype in CRC	(Goel *et al*, 2006)
	?Medulloblastoma			
	? CRC			
Adenovirus	None	E1A	• Binds DNMT1 to increase DNA methyltransferase activity	(Ghosh and Harter, 2003; Turnell *et al*, 2005; Burgers *et al*, 2006; McCabe *et al*, 2006)
			• Binds E2F promoters to demethylate H3K9	
			• Binds and peturbs p300/CBP HAT activity	
*RNA viruses*				
HTLV1/2	ATL	Tax	• Binds with p300/CBP to repress transcription	(Kwok *et al*, 1996)

ATL=adult T cell leukemia; BKV=BK virus; BL=Burkitts Lymphoma; CRC=colorectal cancer; EBV=Epstein–Barr Virus; HBV=hepatitis B virus; HCC=hepatocellular carcinoma; HD=Hodgkins Disease; HPV=human papilloma virus; HTLV=human lymphotrophic virus; JCV=JC virus; KS=Kaposi Sarcoma; KSHV=Kaposi Sarcoma-associated herpesvirus; MCD=multicentric Castleman's Disease; NPC=Nasopharyngeal Carcinoma; PEL=primary effusion lymphoma; PTLD=posttransplant lymphoproliferative disease; SV40=simian virus 40.

**Table 2:** Potential epigenetic functions of selected KSHV proteins and microRNAs

**KSHV Gene**	**Cellular Homolog**	**Activities**	**Epigenetic functions of cellular homologues**	**References**
K2 (vIL6)	IL6	B-cell growth factor, angiogenesis, hematopoiesis	• IL6 regulates expression of DNMT1	(Croonquist and Van Ness, 2005; Hodge *et al*, 2005; Peng *et al*, 2005)
			• IL6 supports the maintenance of p53 promoter methylation	
			• IL6 supports methylation of the RAD23B DNA repair gene	
			• IL6 induces polycomb group gene EZH2	
ORF74 (vGPCR)	GPCR/ IL-8R	Angiogenesis; transformation	• GPCR signaling regulates histone acetylation and gene transcription	(Kang *et al*, 2005)
KSHV miRNAs (mirK1-mirK12)	None yet identified	Transcriptional repression. Some predicted cellular targets include TNF, SOCS3, RAD21 and MAP3K8	• miRNAs (Oncomirs) such as the let-7 family regulate expression of oncogenes	(Cai *et al*, 2005; Esquela-Kerscher and Slack, 2006)
